# Prognostic Impact of Extranodal Organ Burden in Classical Hodgkin Lymphoma with Extranodal Involvement: A Retrospective Cohort Analysis

**DOI:** 10.3390/jcm15156067

**Published:** 2026-08-04

**Authors:** Salih Sertaç Durusoy, Tayfur Toptaş, Derviş Murat Akkurd, Ali Tekbaş, Abdi İbrahim Halil Sönmez, Handan Haydaroğlu Şahin, Vahap Okan

**Affiliations:** 1 Department of Hematology, Ali Osman Sönmez Oncology Hospital, Bursa 16040, Türkiye; 2 Department of Hematology, Faculty of Medicine, Marmara University, İstanbul 34899, Türkiye; 3 Department of Hematology, Mersin City Training and Research Hospital, Mersin 33110, Türkiye; 4 Department of Hematology, Gaziantep City Hospital, Gaziantep 27010, Türkiye; 5 Department of Hematology, Kilis Prof. Dr. Alaeddin Yavaşça State Hospital, Kilis 79090, Türkiye; 6 Department of Hematology, Faculty of Medicine, Gaziantep University, Gaziantep 27310, Türkiye

**Keywords:** classical Hodgkin lymphoma, extranodal disease, extranodal organ burden, prognosis, overall survival, A-HIPI

## Abstract

**Background**: The prognostic significance of extranodal disease in classical Hodgkin lymphoma remains incompletely defined. Although contemporary models such as the Advanced Hodgkin International Prognostic Index (A-HIPI) improve systemic risk stratification, it remains unclear whether outcomes are more closely associated with specific extranodal organ involvement or with the overall burden of extranodal dissemination. **Methods**: This retrospective single-center study included 89 patients with predominantly advanced-stage classical Hodgkin lymphoma, including a minority of high-risk stage II bulky cases, and documented extranodal involvement at diagnosis. Extranodal disease was evaluated according to both organ-specific involvement and extranodal organ burden (EOB), defined as single extranodal organ involvement (single EO) versus involvement of two or more extranodal organs (≥2 EO). Overall survival (OS) and progression-free survival (PFS) were assessed using Kaplan–Meier analysis and Cox proportional hazard models. Risk stratification was examined using both the International Prognostic Score (IPS) and A-HIPI. **Results**: Patients with ≥2 EO had significantly inferior OS compared with those with single EO involvement (5-year OS, 66.8% vs. 96.7%; log-rank *p* = 0.018), whereas the difference in PFS did not reach statistical significance (5-year PFS, 49.6% vs. 73.5%; log-rank *p* = 0.108). A-HIPI-based stratification significantly discriminated OS (5-year OS, 92.2% vs. 78.2%; *p* = 0.001) and showed borderline discrimination for PFS (5-year PFS, 71.5% vs. 57.2%; *p* = 0.053). In the combined analysis, patients with high A-HIPI risk and ≥2 EO had the poorest outcomes, with a 5-year PFS of 34.1% and a 5-year OS of 25.0%. In a parsimonious multivariable Cox model including EOB and A-HIPI-predicted 5-year risk as a continuous variable, ≥2 EO remained associated with inferior OS (HR 3.83, 95% CI 1.34–10.97; *p* = 0.013), while its association with PFS was adverse but not statistically significant (HR 1.83, 95% CI 0.86–3.88; *p* = 0.114). Organ-specific extranodal involvement showed limited and inconsistent associations with survival across A-HIPI- and IPS-defined subgroups. **Conclusions**: In this retrospective single-center cohort, involvement of multiple extranodal organs was associated with inferior OS after adjustment for continuous A-HIPI-predicted risk, whereas its association with PFS did not reach statistical significance. Individual extranodal sites showed no consistent prognostic associations. These exploratory and hypothesis-generating findings suggest that quantitative assessment of extranodal organ burden may complement existing clinical risk measures; however, confirmation in larger, contemporary, externally validated cohorts is required before clinical application.

## 1. Introduction

Hodgkin lymphoma (HL) is a highly curable malignancy; however, a subset of patients with advanced-stage disease continues to experience inferior outcomes despite contemporary treatment strategies. Accurate risk stratification remains essential to identify patients at higher risk of treatment failure and to guide individualized management approaches. The International Prognostic Score (IPS) has long been used for risk assessment in advanced-stage HL, yet its discriminatory performance has diminished in the modern treatment era, prompting the development of refined prognostic models [1,2,3,4].

The Advanced Hodgkin International Prognostic Index (A-HIPI) was recently proposed as an updated risk stratification tool incorporating readily available clinical and laboratory parameters, demonstrating improved prognostic accuracy compared with IPS in several cohorts. Nevertheless, both IPS and A-HIPI primarily reflect systemic disease characteristics and do not fully account for the heterogeneity of extranodal involvement, which is common in advanced-stage HL and has traditionally been considered an adverse prognostic feature [5,6,7,8].

Previous studies have largely evaluated extranodal disease as a binary variable, without addressing whether prognosis is driven by the specific organ involved or by the extent of extranodal dissemination. Emerging evidence suggests that the extent of extranodal dissemination may provide prognostic information beyond organ-specific involvement alone; however, this hypothesis has not been systematically explored within the framework of contemporary prognostic models such as A-HIPI [9,10,11].

In this study, we aimed to evaluate the prognostic relevance of extranodal disease in patients with advanced-stage Hodgkin lymphoma and high-risk stage II disease by disentangling the effects of organ-specific involvement and EOB on overall survival (OS) and progression-free survival (PFS). Specifically, we examined whether the prognostic impact of extranodal organ involvement differs between low- and high-risk A-HIPI groups, and whether EOB provides additional prognostic information within established risk stratification systems, including A-HIPI and IPS.

## 2. Methods

### 2.1. Study Design and Patient Population

This retrospective single-center cohort study was approved by the Gaziantep University Clinical Research Ethics Committee (Decision No: 2020/384, 30 December 2020) and conducted in accordance with the Declaration of Helsinki. Adult patients diagnosed with Hodgkin lymphoma between January 2006 and December 2020 at Gaziantep University Faculty of Medicine, Department of Hematology, were reviewed. Patients were eligible if they had histologically confirmed Hodgkin lymphoma, documented extranodal organ involvement at diagnosis, first-line treatment with curative intent, and sufficient baseline and follow-up data. Patients who did not receive treatment, had nodular lymphocyte-predominant Hodgkin lymphoma, or had incomplete key clinical or outcome data were excluded. Overall, 89 patients were included. The cohort consisted predominantly of stage III–IV disease but also included a small number of stage IIB patients with bulky and/or extranodal disease who were managed according to advanced-stage treatment principles.

### 2.2. Data Collection and Variables

Baseline clinical and laboratory data included age, sex, stage, bulky disease, B symptoms, hemoglobin, absolute lymphocyte count, serum albumin, and lactate dehydrogenase. Organ-specific criteria for extranodal involvement were defined as follows: bone marrow involvement by biopsy and/or PET uptake consistent with infiltration; bone involvement by PET-positive lesions with or without CT/MRI findings; lung involvement by parenchymal and/or pleural lesions on imaging; and liver involvement by focal PET uptake and/or radiologic lesions. Other extranodal sites were recorded as specified in the dataset. All assessments were based on available clinical reports. Equivocal findings were not counted as definite extranodal involvement unless supported by additional clinical, imaging, or histopathologic evidence. In the present study, extranodal organ burden (EOB) refers to the number of extranodal organs involved at diagnosis rather than the volumetric extent of disease. EOB was classified as single extranodal organ involvement (single EO) versus involvement of two or more extranodal organs (≥2 EO). Direct contiguous extension from adjacent nodal disease was not classified as extranodal organ involvement. Only distinct extranodal organ sites recorded in the institutional database were included in the analysis. Extranodal organ burden was categorized in this way to distinguish limited extranodal extension from more disseminated extranodal disease and to preserve statistical stability given the modest sample size and small numbers in higher organ-count categories. This cutoff was not derived using ROC analysis or outcome-based optimization. Analysis of the exact number of involved extranodal organs as a continuous or ordinal variable was considered; however, the small number of patients and events in the higher organ-count categories was expected to yield unstable estimates.

### 2.3. Risk Stratification

Prognostic risk was assessed using both the International Prognostic Score (IPS) and the Advanced Hodgkin International Prognostic Index (A-HIPI). IPS was categorized as low risk (<4) and high risk (≥4) according to conventional thresholds. A-HIPI was calculated at diagnosis using predefined clinical and laboratory variables. In addition, A-HIPI-based predicted 5-year overall survival (OS) and progression-free survival (PFS) probabilities were derived for each patient. For survival analyses, patients were dichotomized into low- and high-risk groups using ROC-derived cutoffs. Cutoffs based on predicted 5-year PFS were used for PFS analyses, whereas cutoffs based on predicted 5-year OS were used for OS analyses.

ROC-derived cutoffs were used only for exploratory within-cohort stratification and were not intended to represent validated clinical thresholds. Because these cutoffs were derived and evaluated in the same dataset, this approach may introduce optimism bias and limit generalizability. Therefore, in the primary multivariable analysis, A-HIPI was modeled as a continuous predictor rather than relying on internally derived categorical thresholds.

### 2.4. Treatment and Response Assessment

All patients received ABVD (doxorubicin, bleomycin, vinblastine, and dacarbazine) as first-line chemotherapy. No patients received brentuximab vedotin-containing regimens or escalated BEACOPP during first-line treatment. Consolidative radiotherapy was administered to 13 patients (14.6%), primarily in the presence of bulky or residual disease, according to treating physician discretion and contemporary institutional practice. Treatment response was assessed by PET/CT. Given the long inclusion period (2006–2020), PET/CT availability and standardization were not uniform across all patients, particularly in earlier years of the cohort, and response assessments were therefore based on available imaging data within routine clinical practice. PET responses were interpreted using the Deauville 5-point scale, and final response categories were assigned according to the Lugano classification.

### 2.5. Outcomes

The primary endpoints were overall survival (OS) and progression-free survival (PFS). OS was defined as the time from diagnosis to death from any cause or last follow-up. PFS was defined as the time from diagnosis to progression, relapse, death, or last follow-up, whichever occurred first. Patients without an event were censored at the date of last clinical contact. Median follow-up was planned to be estimated using the reverse Kaplan–Meier method.

### 2.6. Statistical Analysis

Statistical analyses were performed using R 4.5.0 (R Foundation for Statistical Computing, Vienna, Austria). Continuous variables were summarized as median and interquartile range (IQR), whereas categorical variables were presented as frequency and percentage. Survival curves were estimated using the Kaplan–Meier method and compared using the log-rank test. Univariable Cox proportional hazards regression analyses were performed to evaluate associations between extranodal organ involvement patterns and survival outcomes. Given the limited number of outcome events, subgroup and supplementary multivariable analyses were considered exploratory and interpreted cautiously. A parsimonious multivariable Cox model was constructed including EOB and A-HIPI–predicted 5-year risk as a continuous variable. The primary multivariable model was intentionally restricted because of the modest number of outcome events. EOB was included as the main exposure of interest, and A-HIPI-predicted 5-year risk was modeled as a continuous covariate to account parsimoniously for baseline prognostic information without relying on internally derived categorical thresholds. Additional variables were not entered into the primary model to reduce the risk of model overfitting; therefore, residual confounding cannot be excluded. An additional exploratory model including A-HIPI risk group was analyzed separately because of potential overlap between prognostic variables. Results were reported as hazard ratios (HRs) with 95% confidence intervals (CIs). All statistical tests were two-sided, and a *p*-value <0.05 was considered statistically significant.

### 2.7. Ethical Considerations

The study was conducted in accordance with the Declaration of Helsinki and was approved by the local institutional ethics committee. Given the retrospective design and the use of anonymized patient data, the requirement for informed consent was waived in accordance with local regulations.

## 3. Results

### 3.1. Patient Characteristics and Extranodal Disease Distribution

A total of 89 patients with Hodgkin lymphoma and documented extranodal involvement at diagnosis were included in the analysis. The median age was 33 years (IQR, 26–49), and 48 patients (53.9%) were male. Most patients had stage IV disease (65.2%), whereas 21.3% had stage III and 13.5% had stage II with bulky disease. Bulky disease was present in 17 patients (19.1%). According to the International Prognostic Score, 64 patients (71.9%) were classified as low risk (IPS <4) and 25 (28.1%) as high risk (IPS ≥4). Thirteen patients (14.6%) received consolidative radiotherapy in addition to ABVD, whereas the remaining patients were treated with ABVD alone. Baseline clinical and laboratory characteristics, along with A-HIPI-based risk group distributions, are summarized in Table 1.

Extranodal involvement showed a heterogeneous distribution across organ sites. Bone marrow (44.9%) and lung (41.6%) were the most frequently involved sites, followed by bone (22.5%) and liver (20.2%), whereas other extranodal sites were identified in 7.9% of patients. Single EO involvement was observed in 60 patients (67.4%), while 29 patients (32.6%) had involvement of ≥2 EO at diagnosis (Table 1).

### 3.2. Extranodal Organ Burden and Survival Outcomes

**Figure 1:**
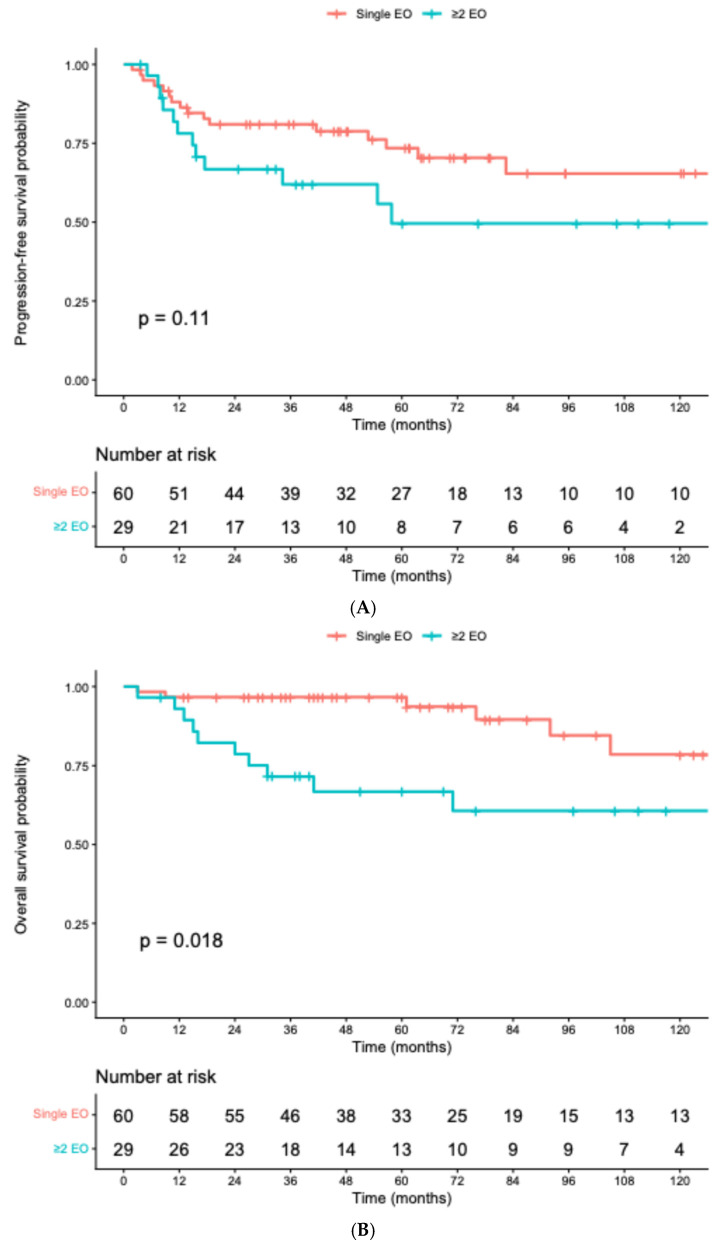
Kaplan–Meier estimates of (**A**) progression-free survival and (**B**) overall survival according to extranodal organ burden in patients with advanced-stage Hodgkin lymphoma. Single EO denotes single extranodal organ involvement; ≥2 EO, involvement of two or more extranodal organs.

Median follow-up, estimated using the reverse Kaplan–Meier method, was 70 months. Seventeen deaths were recorded. Deaths occurred in 7 patients with single EO involvement and in 10 patients with involvement of ≥2 EO. Progression events were observed in 16 and 12 patients, respectively (Table 2; Figure 1A).

Progression-free survival did not differ significantly according to EOB. Median PFS was not reached in the single EO group and was 57.8 months (95% CI, 34.3–NR) in the ≥2 EO group (log-rank *p* = 0.108). The corresponding 5-year PFS rates were 73.5% and 49.6%, respectively.

Overall survival differed significantly according to EOB (log-rank *p* = 0.018). The 5-year OS rate was lower in patients with involvement of ≥2 EO than in those with single EO involvement (66.8% vs. 96.7%). Median OS estimates should be interpreted cautiously because of sparse late events and limited numbers of patients remaining at risk in the tail of the Kaplan–Meier curves; therefore, fixed-time survival estimates were emphasized over median OS values (Figure 1B).

### 3.3. A-HIPI Risk Groups and Survival Outcomes

**Figure 2:**
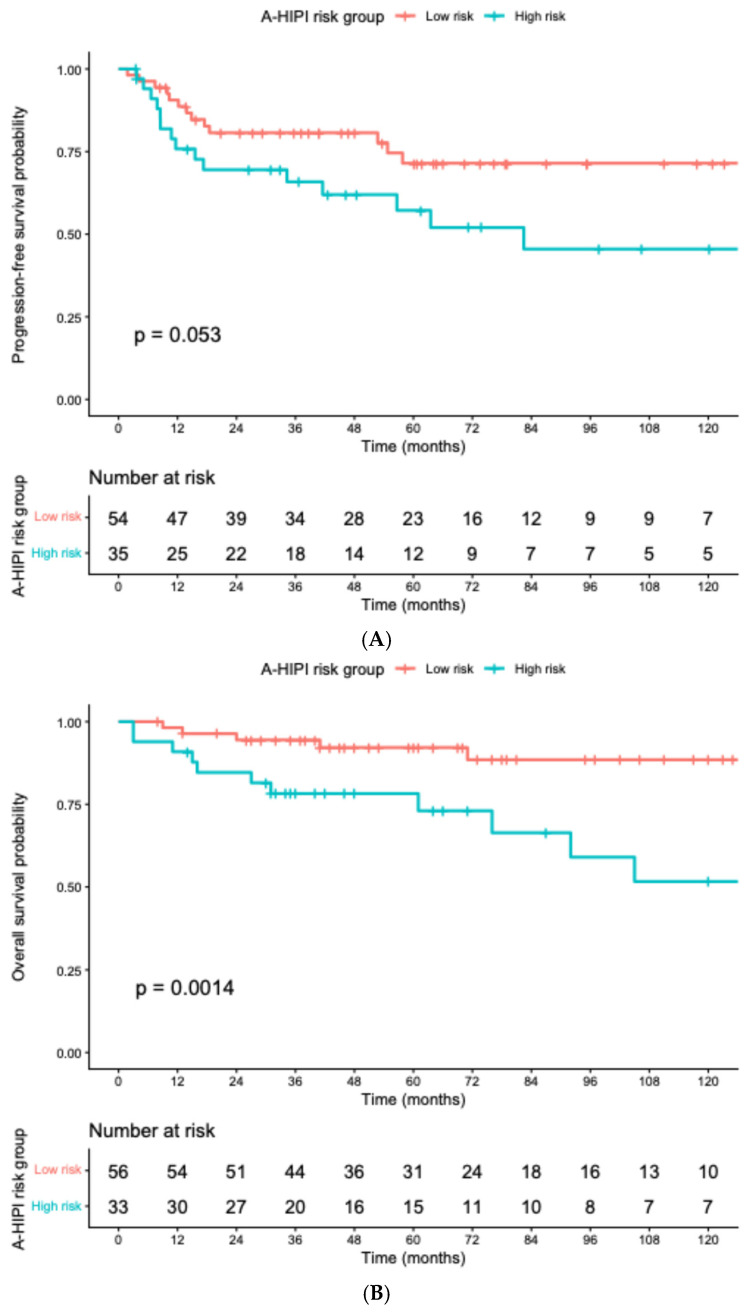
(**A**) Progression-free survival and (**B**) overall survival curves according to A-HIPI risk groups. Patients were stratified into low-risk and high-risk groups using endpoint-specific ROC-derived A-HIPI cutoffs for PFS and OS.

Patients were stratified into low- and high-risk groups using ROC-derived A-HIPI cutoffs for predicted 5-year PFS and OS. In the PFS analysis, 54 patients were classified as low risk and 35 as high risk, with 13 and 15 progression events, respectively. Median PFS was not reached in the low-risk group and was 82.5 months (95% CI, 41.5–NR) in the high-risk group. The corresponding 5-year PFS rates were 71.5% and 57.2%, and the between-group difference did not reach statistical significance (log-rank *p* = 0.053) (Table 3, Figure 2A).

For the OS analysis, 56 patients were classified as low risk and 33 as high risk. Deaths occurred in 5 and 12 patients, respectively. Median OS was not reached in the low-risk group and was 149.0 months (95% CI, 76.0–NR) in the high-risk group. The 5-year OS rates were 92.2% and 78.2%, respectively. OS was significantly better in the low-risk group than in the high-risk group (log-rank *p* = 0.001) (Table 3, Figure 2B).

### 3.4. Combined Effect of A-HIPI Risk Group and Extranodal Organ Burden

**Figure 3:**
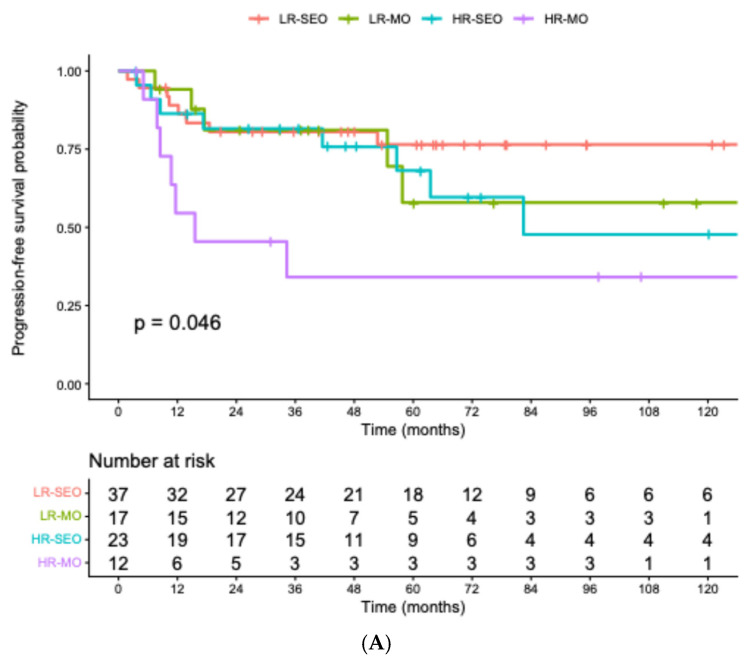

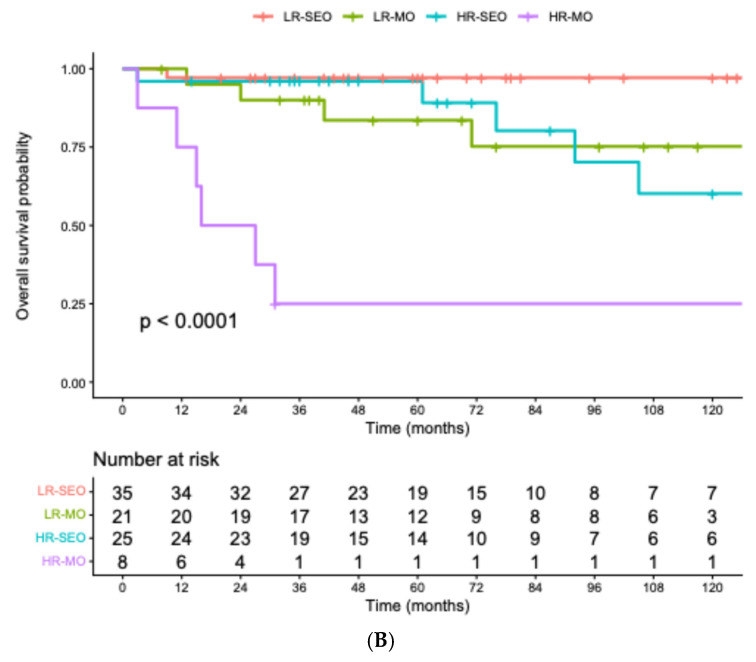
(**A**) Progression-free and (**B**) overall survival according to combined A-HIPI risk group and extranodal organ burden. LR-SEO denotes low A-HIPI risk with single extranodal organ involvement; LR-MO, low A-HIPI risk with two or more extranodal organ involvement; HR-SEO, high A-HIPI risk with single extranodal organ involvement; and HR-MO, high A-HIPI risk with two or more extranodal organ involvement.

Patients were further stratified according to combined ROC-derived A-HIPI risk group and EOB (Table 4). For PFS, outcomes differed significantly across the four groups (log-rank *p* = 0.046), with the poorest results observed in patients with high A-HIPI risk and involvement of ≥2 EO (median PFS, 15.6 months; 95% CI, 10.7–NR; 5-year PFS, 34.1%) (Table 4; Figure 3A). In Cox analysis, only this subgroup showed a significantly increased risk of progression compared with the low-risk single EO reference group (HR 3.95, *p* = 0.008).

For OS, survival also differed significantly across groups (log-rank *p* < 0.001) (Table 4; Figure 3B). Again, the high-risk subgroup with involvement of ≥2 EO had the poorest outcome (median OS, 21.5 months; 95% CI, 15.0–NR; 5-year OS, 25.0%), whereas the low-risk single EO subgroup had the most favorable survival (5-year OS, 97.1%). In the exploratory Cox analysis, the high A-HIPI risk/≥2 EO subgroup showed the greatest estimated risk of death relative to the low-risk/single EO reference group (HR 49.10, *p* < 0.001). However, this estimate was based on only eight patients and six deaths in the high-risk/≥2 EO subgroup, together with only one death in the reference group, and should therefore be interpreted with substantial caution because of sparse events and imprecision.

### 3.5. Parsimonious Multivariable Cox Regression Analysis for Overall Survival and Progression-Free Survival

In a parsimonious multivariable Cox regression model including EOB and A-HIPI-predicted 5-year risk as a continuous variable, involvement of ≥2 EO was associated with inferior overall survival after adjustment for A-HIPI-predicted risk (HR 3.83; 95% CI, 1.34–10.97; *p* = 0.013). For progression-free survival, ≥2 EO showed an adverse but non-significant association in the adjusted model (HR 1.83; 95% CI, 0.86–3.88; *p* = 0.114). A-HIPI-predicted risk was not significantly associated with either overall survival (HR 0.97; 95% CI, 0.93–1.01; *p* = 0.127) or progression-free survival (HR 0.99; 95% CI, 0.94–1.04; *p* = 0.780) (Table 5).

### 3.6. Additional Exploratory Analyses

Across A-HIPI risk groups, organ-specific extranodal involvement did not show a uniform association with OS and PFS. In the low A-HIPI group, bone marrow involvement was associated with inferior PFS (HR 3.39; 95% CI, 1.14–10.07; *p* = 0.028), whereas involvement of other extranodal sites was associated with inferior OS (HR 13.74; 95% CI, 2.48–76.13; *p* = 0.003). Other organ-specific sites, including bone, lungs, and livers, were not significantly associated with OS or PFS in this subgroup. In the high A-HIPI group, no individual organ-specific extranodal site showed a statistically significant association with either OS or PFS.

Similarly, among patients with high IPS (≥4), bone marrow, bone, lung, and liver involvement were not significantly associated with OS, whereas involvement of other extranodal sites was associated with worse OS (HR 11.31; 95% CI, 1.03–124.83; *p* = 0.048). No organ-specific extranodal site was significantly associated with PFS in the high-IPS subgroup.

An exploratory multivariable model incorporating A-HIPI risk group together with EOB suggested that extranodal burden remained associated with both OS and PFS. However, the estimated effect of A-HIPI was not fully concordant with the univariable survival analyses, and confidence intervals were unstable, indicating possible overlap among prognostic variables and limited event numbers. Accordingly, these supplementary analyses should be regarded as hypothesis-generating and interpreted with caution due to small subgroup sizes, multiple comparisons, and the resulting risk of unstable estimates and false-positive findings.

## 4. Discussion

In advanced-stage classical Hodgkin lymphoma, prognostic models have evolved from the historical IPS-7 to the simplified IPS-3 and, more recently, to the A-HIPI model in an effort to improve risk discrimination in the modern treatment era [5]. However, these models are primarily based on baseline clinical and laboratory variables, and extranodal organ involvement has not been incorporated into prognostic scoring systems [12,13]. This may be clinically relevant, as extranodal dissemination is unlikely to be biologically homogeneous, and involvement of multiple extranodal organs may identify a distinct subgroup with more extensive disease dissemination and adverse survival risk that is not fully captured by IPS- or A-HIPI-based prognostic stratification. Involvement of multiple extranodal organs may reflect more extensive disease dissemination and a higher overall disease burden, which may partly explain its association with poorer outcomes. However, this biological interpretation remains hypothetical. Therefore, in the present study, we investigated the prognostic impact of extranodal organ count in patients with extranodal Hodgkin lymphoma and examined its relationship with established risk models, particularly IPS and A-HIPI [14,15,16,17].

Yang et al. demonstrated that primary extranodal classical Hodgkin lymphoma was associated with a higher recurrence rate than nodal disease (36.4% vs. 13.1%, *p* = 0.003), as well as inferior 5-year OS (64.6% vs. 97.7%, *p* = 0.001) and PFS (42.4% vs. 82.2%, *p* < 0.001) [18]. Consistent with these observations, our data suggest that involvement of a greater number of extranodal organs is associated with worse outcomes. Patients with involvement of ≥2 EO had significantly inferior overall survival compared with those with single EO involvement (log-rank *p* = 0.018), with 5-year OS rates of 66.8% and 96.7%, respectively. Moreover, in the parsimonious multivariable model, involvement of ≥2 EO remained independently associated with inferior overall survival (HR 3.83; 95% CI, 1.34–10.97; *p* = 0.013) and showed an adverse association with progression-free survival (HR 1.83; 95% CI, 0.86–3.88; *p* = 0.114). Although the unadjusted Kaplan–Meier comparison for PFS did not reach statistical significance (log-rank *p* = 0.108), patients with ≥2 EO had a numerically lower 5-year PFS rate compared with those with single EO (49.6% vs. 73.5%).

In the original HoLISTIC development and validation study, A-HIPI demonstrated better prognostic performance than the historical IPS, with optimism-corrected c-statistics of 0.590 for 5-year PFS and 0.720 for 5-year OS [5]. More recently, external validation in a Turkish single-center cohort of 207 patients also confirmed that A-HIPI remained prognostic for both endpoints, with C-indices of 0.605 for PFS and 0.740 for OS [13].

In our dataset, A-HIPI-based stratification significantly discriminated OS, with 5-year OS rates of 92.2% in the low-risk group and 78.2% in the high-risk group (*p* = 0.001), whereas the separation for PFS was more modest and borderline significant (5-year PFS, 71.5% vs. 57.2%; *p* = 0.053).

The weaker discrimination of A-HIPI for PFS in our cohort may reflect the relatively small and clinically selected nature of the study population, enrichment for stage IV and multi-organ extranodal disease, and the growing impact of dynamic treatment-related factors such as PET-guided response assessment that are not fully captured by baseline prognostic models [12,13,19,20,21,22].

Importantly, prognostic separation appeared more pronounced when A-HIPI was considered together with EOB. In the combined four-group analysis, patients with high A-HIPI risk and involvement of ≥2 EO had the poorest observed outcomes, with a 5-year PFS of 34.1% and a 5-year OS of 25.0%. However, these findings should be interpreted cautiously because the analysis was exploratory and based on small subgroups with sparse events. In particular, the markedly elevated hazard ratio estimate for mortality in the high-risk/≥2 EO group was based on only eight patients and six deaths, compared with one death in the low-risk/single EO reference group, and therefore likely reflects substantial imprecision. Thus, the combined analysis should be viewed as evidence of risk separation within this cohort rather than as a precise estimate of effect magnitude.

Previous studies have likewise suggested limited prognostic relevance of specific extranodal sites. Ma et al. reported no significant survival differences according to liver, lung, bone, or other extranodal involvement, and IPS also failed to discriminate outcome (5-year OS: 90.8% vs. 87.3%, *p* = 0.504; 5-year DFS: 89.5% vs. 84.1%, *p* = 0.324) [23]. Similarly, Li et al. found no significant association between IPS and survival across different extranodal sites, despite numerically higher 5-year OS and DFS rates in the low-risk group (OS: 93.3% vs. 83.3%, *p* = 0.841; DFS: 75.0% vs. 73.8%, *p* = 0.841) [24].

In line with this literature, our organ-specific supplementary analyses should be interpreted cautiously. Most site-specific associations were not statistically significant across A-HIPI- or IPS-defined subgroups. In the low A-HIPI group, bone involvement was not associated with OS or PFS (HR 2.52, *p* = 0.111; HR 1.52, *p* = 0.453), and lung involvement was likewise non-significant for both endpoints (HR 1.56, *p* = 0.454; HR 0.54, *p* = 0.237). Similarly, in patients with high IPS, liver involvement was not associated with OS or PFS (HR 0.90, *p* = 0.907; HR 1.04, *p* = 0.960), and bone involvement also remained non-significant for both OS and PFS (HR 3.88, *p* = 0.229; HR 3.76, *p* = 0.281).

In multivariable analyses, EOB remained independently associated with outcome. In the parsimonious model including EOB and A-HIPI-predicted 5-year risk as a continuous variable, involvement of ≥2 EO was associated with inferior overall survival (HR 3.83; 95% CI, 1.34–10.97; *p* = 0.013), while showing an adverse but non-significant association with progression-free survival (HR 1.83; 95% CI, 0.86–3.88; *p* = 0.114).

The effect estimates for A-HIPI were attenuated and not statistically significant for either overall survival (HR 0.97; 95% CI, 0.93–1.01; *p* = 0.127) or progression-free survival (HR 0.99; 95% CI, 0.94–1.04; *p* = 0.780). Given the limited number of events and potential overlap among prognostic variables, these findings should be interpreted with caution. Additional exploratory analyses incorporating alternative model specifications yielded unstable estimates with wide confidence intervals and are therefore considered hypothesis-generating.

This study has several limitations. Its retrospective design introduces the possibility of selection bias, incomplete data capture, and residual confounding. The relatively small sample size and limited number of events, particularly in subgroup and organ-specific analyses, reduce statistical precision and increase the risk of unstable effect estimates. In addition, the long inclusion period (2006–2020) may have introduced temporal heterogeneity in diagnostic imaging, staging procedures, PET/CT availability, response assessment practices, supportive care, and salvage treatment strategies. These changes may have affected outcome estimation and limit direct comparability across patients treated in different time periods. All patients in this cohort received ABVD-based first-line therapy; therefore, the applicability of these findings to contemporary treatment strategies, including PET-adapted approaches and newer frontline regimens, remains uncertain.

Given the limited number of events, multivariable analyses were restricted to parsimonious models, and the results should be interpreted cautiously. In addition, A-HIPI-based risk stratification relied on ROC-derived cutoffs generated within the same dataset, which may introduce optimism bias and limit generalizability.

Because relatively few patients remained at risk during late follow-up, median survival estimates and the tail behavior of Kaplan–Meier curves should be interpreted with caution. Therefore, fixed-time survival estimates, particularly 5-year OS and PFS rates, were emphasized as more clinically informative measures.

Organ-specific analyses were exploratory and based on small subgroup sizes, especially for less frequent sites, increasing the risk of unstable estimates and false-positive findings. Accordingly, these results should be considered hypothesis-generating.

Diagnostic definitions of extranodal involvement were based on retrospective assessment of radiologic and/or histopathologic data, and heterogeneity in imaging modalities, PET/CT availability, and interpretation over the long study period may have introduced some degree of misclassification, particularly for borderline radiologic or PET-based findings and in distinguishing true organ involvement from contiguous extension.

Because the cohort was restricted to patients with extranodal disease, the findings may not be directly generalizable to the broader population of patients with advanced-stage Hodgkin lymphoma. Although the cohort was predominantly composed of stage III/IV patients, a small number of high-risk stage II cases were pragmatically included because they had bulky and/or extranodal disease requiring treatment approaches like those used in advanced-stage Hodgkin lymphoma.

Despite these limitations, the study also has important strengths. It addresses a clinically relevant and insufficiently explored question by distinguishing the extent of extranodal disease from site-specific involvement. It evaluates EOB within the frameworks of both IPS and A-HIPI and shows that a simple dissemination-based classification according to the number of involved extranodal organs can identify higher-risk patients even when systemic risk models appear relatively favorable. The consistent association between multiorgan extranodal involvement and inferior outcomes across univariable, combined group, and multivariable analyses supports the overall findings of the study.

## 5. Conclusions

In conclusion, in this retrospective single-center cohort of patients with advanced-stage or high-risk stage II classical Hodgkin lymphoma and extranodal involvement, multiorgan extranodal disease was associated with inferior overall survival and showed an adverse, although not statistically significant, association with progression-free survival in the adjusted analysis. Individual extranodal sites did not demonstrate consistent prognostic associations, although these exploratory analyses were limited by small subgroup sizes. These findings suggest that the extent of extranodal organ involvement, as measured by extranodal organ burden, may provide additional prognostic information beyond baseline clinical risk models; however, external validation in larger, uniformly staged and treated cohorts is required before extranodal burden can be formally integrated into contemporary prognostic models.

## Figures and Tables

**Table 1:** Baseline Characteristics of Patients with Advanced-Stage or High-Risk Stage II Classical Hodgkin Lymphoma and Extranodal Involvement (*n* = 89).

Characteristic	Value
Age, median (IQR), years	33 (26–49)
Male, n (%)	48 (53.9)
Disease stage, n (%)	
Stage II Bulky	12 (13.5)
Stage III	19 (21.3)
Stage IV	58 (65.2)
A-HIPI	
PFS high-risk, n (%)	35 (39.3)
OS high-risk, n (%)	33 (37.1)
Bulky disease, n (%)	17 (19.1)
High-risk International Prognostic Score (IPS), n (%)	25 (28.1)
Baseline laboratory parameters	
Hemoglobin, g/dL, median (IQR)	11.3 (9.7–13.2)
Lymphocyte count, ×10^9^/L, median (IQR)	1.5 (1.0–2.0)
LDH, U/L, median (IQR)	259 (205–323)
Serum albumin, g/dL, median (IQR)	3.8 (3.3–4.1)
Extranodal organ involvement, n (%)	
Bone marrow	40 (44.9)
Lung	37 (41.6)
Bone	20 (22.5)
Liver	18 (20.2)
Other sites	7 (7.9)
Number of extranodal organs ≥2, n (%)	29 (32.6)

IQR denotes interquartile range; LDH, lactate dehydrogenase; A-HIPI, advanced-stage Hodgkin lymphoma international prognostic index.

**Table 2:** PFS and OS according to EOB.

Outcome	Single EO	≥2 EO	*p*-Value
Progression-free survival (PFS)			
Patients, n	60	29	
Events, n	16	12	
Median PFS, months (95% CI)	NR	57.8 (34.3–NR)	0.108
5-year PFS, %	73.5%	49.6%	
Overall survival (OS)			
Patients, n	60	29	
Events, n	7	10	
Median OS, months (95% CI)	149.0 (149.0–NR)	NR	0.018
5-year OS, %	96.7%	66.8%	—

EOB denotes extranodal organ burden; OS, overall survival; PFS, progression-free survival; CI, confidence interval; NR, not reached.

**Table 3:** Survival outcomes according to ROC-derived A-HIPI risk groups.

Outcome	Low-Risk	High-Risk	*p*-Value
Progression-free survival (PFS)			
Patients, n	54	35	
Events, n	13	15	
Median PFS, months (95% CI)	NR	82.5 (41.5–NR)	0.053
5-year PFS, %	71.5%	57.2%	
Overall survival (OS)			
Patients, n	56	33	
Events, n	5	12	
Median OS, months (95% CI)	NR	149.0 (76.0–NR)	0.001
5-year OS, %	92.2%	78.2%	—

ROC denotes Receiver Operating Characteristic; OS, overall survival; PFS, progression-free survival; CI, confidence interval; NR, not reached.

**Table 4:** PFS and OS according to combined ROC-derived A-HIPI risk groups and EOB.

Outcome	LR-Single EO	LR- ≥2 EO	HR-Single EO	HR- ≥2 EO	*p*-Value
Progression-free survival (PFS)					
Patients, n	37	17	23	12	
Events, n	8	5	8	7	
Median PFS, months (95% CI)	NR	NR	82.5 (56.6–NR)	15.6 (10.7–NR)	0.046
5-year PFS, %	76.5	57.9	68.2	34.1	
Overall survival (OS)					
Patients, n	35	21	25	8	
Events, n	1	4	6	6	
Median OS, months (95% CI)	NR	NR	149 (92.0–NR)	21.5 (15.0–NR)	<0.001
5-year OS, %	97.1	83.6	96.0	25.0	—

LR-Single EO denotes low-risk and single extranodal organ involvement; LR- ≥2 EO, low-risk and involvement of two or more extranodal organs; HR-Single EO, high-risk and single extranodal organ involvement; HR- ≥2 EO, high-risk and involvement of two or more extranodal organs; ROC, Receiver Operating Characteristic; OS, overall survival; PFS, progression-free survival; CI, confidence interval; NR, not reached.

**Table 5:** Parsimonious multivariable Cox regression analysis for OS and PFS.

	OS		PFS	
Outcome	HR (95% CI)	*p*	HR (95% CI)	*p*
A-HIPI predicted 5-year risk, continuous	0.97 (0.93–1.01)	0.127	0.99 (0.94–1.04)	0.780
≥2 EO vs. single EO	3.83 (1.34–10.97)	0.013	1.83 (0.86–3.88)	0.114

A-HIPI denotes Advanced-stage Hodgkin Lymphoma International Prognostic Index; EOB, extranodal organ burden; EO, extranodal organ; OS, overall survival; PFS, progression-free survival; HR, hazard ratio; CI, confidence interval.

## Data Availability

The data presented in this study are available on request from the corresponding author.
